# Sputum availability and quality in country-level TB prevalence surveys

**DOI:** 10.5588/ijtldopen.24.0117

**Published:** 2024-11-01

**Authors:** P. Papadopoulou, M. Gaeddert, A. Gupta-Wright, C.M. Denkinger, F.M. Marx

**Affiliations:** ^1^ Department of Infectious Disease and Tropical Medicine, Heidelberg University Hospital, Heidelberg, Germany;; ^2^ Department of Infectious Diseases, North Bristol NHS Trust, Bristol, UK;; ^3^ Department of Infectious Diseases, Imperial College London, London, UK;; ^4^ German Center for Infection Research (DZIF), Partner Site Heidelberg, Heidelberg, Germany;; ^5^ DSI-NRF South African Centre of Excellence in Epidemiological Modelling and Analysis (SACEMA), Faculty of Science, Stellenbosch University, Stellenbosch, South Africa.

**Keywords:** tuberculosis, people living with HIV, Xpert MTB/RIF, national-level TB surveys

Dear Editor,

TB remains a leading cause of death worldwide.^1^ Improving TB detection globally is vital to reduce transmission, disease burden, morbidity and mortality. Currently, bacteriological confirmation of TB requires that individuals produce a sputum sample for testing. Sputum scarcity, defined as the inability to produce an adequate sputum sample is a known challenge, but its frequency among people accessing diagnostic services is not well established. Previous studies have shown that sputum scarcity is high in certain groups, such as people living with HIV (PLWH) and children.^2,3^ An individual participant data meta-analysis of TB diagnostic yield among PLWH found that, 1,842 (18%) of 10,202 participants were unable to produce sputum.^4^ The inability to produce a sputum sample may also affect TB prevalence surveys, which typically rely on sputum-based bacteriological confirmation among eligible survey participants to obtain estimates of TB prevalence. Current country-level TB prevalence surveys usually focus on individuals aged ≥15 years who are eligible for sputum testing conditional on a positive initial screening test for either TB-characteristic symptoms and/or an abnormal chest radiography. WHO recommends that in TB prevalence surveys, two sputum samples per participant are collected: one on the spot, and a second on the morning of the following day. For participants of TB prevalence surveys who are unable to produce sputum, or who do not provide a sputum sample for other reasons, missing value imputations is recommended to reduce bias in the overall TB prevalence estimate.^5^ To maximise sample availability, WHO emphasises that even salivary samples should be accepted for participants who struggle to produce a mucous or purulent sample.^6^ The extent to which sputum samples are unavailable or of poor quality in country-level TB prevalence surveys has not been reported to date.

We reviewed published articles and reports of country-level TB prevalence surveys conducted in high-burden countries since 2000, based on country lists published by the WHO (Fig. 2.3.1 National surveys of the prevalence of TB disease, actual (2000–2021) and planned (2022))^7^ to estimate the proportion of TB prevalence survey participants eligible for sputum testing for whom a sputum sample was not available (for any reason). We investigated how availability varied in high-burden countries and subgroups of participants, and attempted to determine the reasons for the lack of availability. We also reviewed the reports for data on the quality of sputum obtained. We searched PubMed and online sources, including websites of national health authorities, for publications on national TB prevalence surveys. We chose the most recent report if multiple TB prevalence surveys were conducted in the same country. We extracted data on the number of screened participants, eligible participants, and those submitting sputum samples. If reported, we also extracted information about reasons for not submitting sputum and about the quality of sputum submitted.

We identified 30 reports of country-level TB prevalence surveys conducted in high-burden countries and published in English: 17 were conducted in Africa and 13 in Asia. A total of 293,430 adult survey participants were eligible for sputum testing, of whom 22,369 (7.6%) did not provide a sputum sample for any reason. The percentage of survey participants who did not provide sputum varied between 0.2% in Myanmar and 18.6% in Pakistan (Figure). We found information on sputum unavailability in subgroups of participants in 7 out of 30 reports. Reports indicated that sputum unavailability was higher in younger participants (e.g., aged 15–34 years), those living in urban areas, and those with higher economic status. Five reports included data on reasons for not submitting sputum samples: the most common ones included the participants’ sputum scarcity, refusal to provide samples, absence during sputum collection and recording errors.

**Figure.**
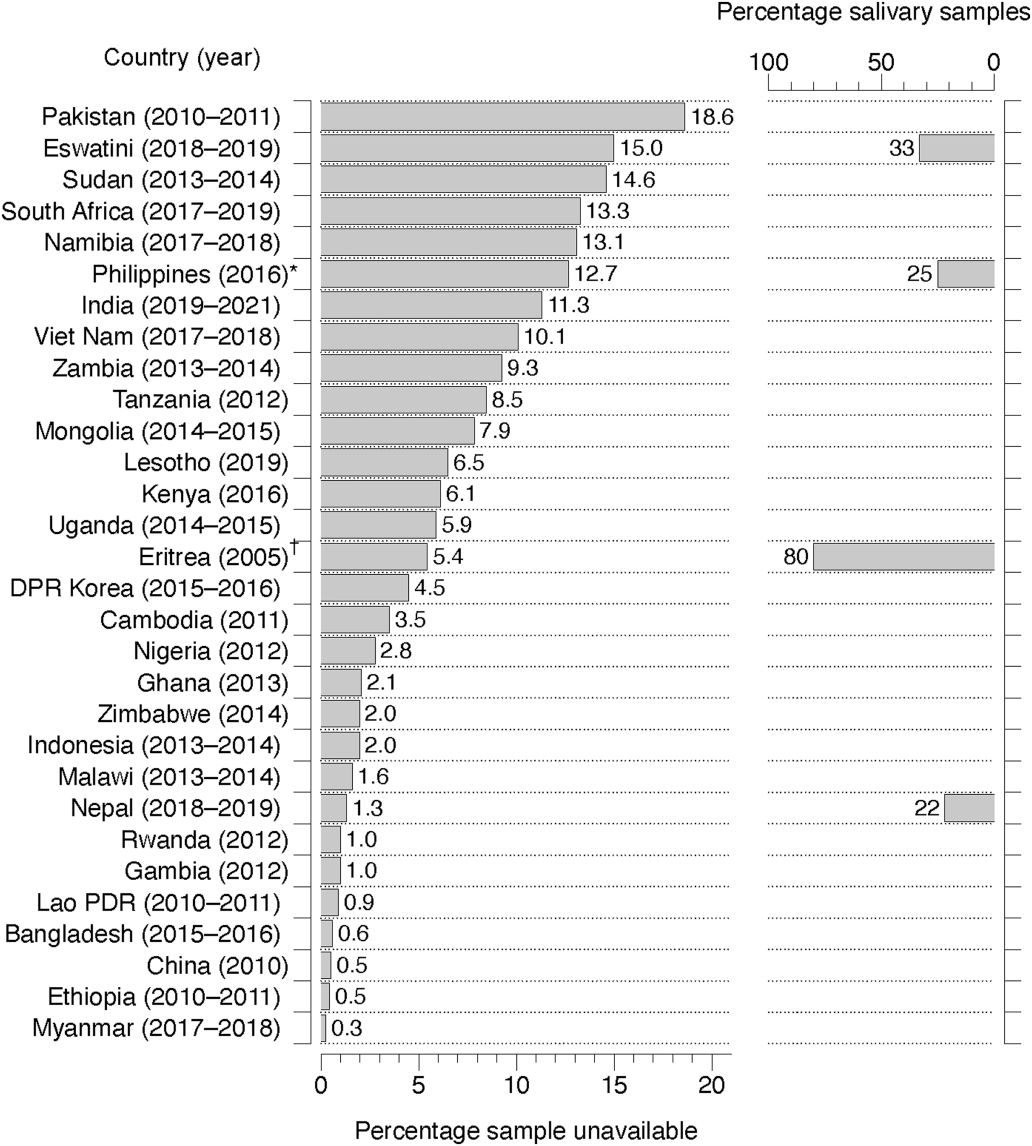
Proportions of unavailable sputum samples and low-quality (salivary) samples in country-level TB prevalence surveys in high TB burden countries. *Xpert-positive sputum results. ^†^Spot samples. DPR = Democratic People’s Republic of Korea; PDR = Lao People’s Democratic Republic.

An assessment of sputum quality through visual exploration was mentioned under sputum collection methods in 17 out of 30 reports. However, only 4 included data about the quality of sputum (Figure). The proportion of samples judged as salivary was 22% in Nepal, 33% in Eswatini, with 80% for spot and 90% for morning samples in Eritrea. In the Philippines, sputum quality was reported only for positive samples, with 25% of samples positive on Xpert^®^ MTB/RIF (Cepheid, Sunnyvale, CA, USA) reported as salivary. In Eritrea, the diagnostic yield of sputum smear-positive TB was five times higher in specimens categorised as ‘mucopurulent sputum’ compared to saliva (0.25% vs. 0.05%). None of the 30 TB prevalence surveys reported exclusion of salivary samples from their assessment of TB prevalence.

Although sputum unavailability appears to be generally low in country-level TB prevalence surveys, there is considerable variation between countries. Younger age groups, participants in urban settings and those with higher socio-economic status appear less likely to submit sputum samples. Reasons for unavailable samples were inconsistently reported; it is therefore not possible to determine the frequency of sputum scarcity in TB prevalence surveys. Data suggest that low-quality samples (i.e., samples appearing visually as saliva) are common, and that the TB diagnostic yield might be considerably lower in these samples (reported for one country). These findings are consistent with a study of sputum quality in a sub-national-level TB prevalence survey conducted in central India,^8^ a study of active TB case-finding among prison inmates in Brazil,^9^ and an operational research study in Kenya,^10^ which showed that saliva samples were common especially among participants not reporting TB symptoms,^8,9^ and associated with lower Xpert positivity. We speculate that the high availability of sputum samples reported in country-level TB prevalence surveys could be linked to reduced sputum quality, as lower-quality samples (e.g. saliva) are accepted for testing. As sample quality is likely to affect diagnostic sensitivity,^8–12^ we emphasise that poor sputum quality might lead to underestimation of TB prevalence. Moreover, it might affect yield in community-based active case-finding interventions.

WHO guidance for national TB prevalence surveys highlights the importance of good-quality sputum samples,^6^ but sputum quality is only infrequently documented. We recommend that the results of sputum quality and their differential yield be included in TB prevalence surveys. Additional efforts are warranted to determine the role of sputum scarcity and poor-quality sputum samples in country-level TB prevalence surveys.
